# Correction: Godugu et al. Anti-Cancer Activities of Thyrointegrin α_v_β_3_ Antagonist Mono- and Bis-Triazole Tetraiodothyroacetic Acid Conjugated via Polyethylene Glycols in Glioblastoma. *Cancers* 2021, *13*, 2780

**DOI:** 10.3390/cancers14215371

**Published:** 2022-10-31

**Authors:** Kavitha Godugu, Mehdi Rajabi, Shaker A. Mousa

**Affiliations:** The Pharmaceutical Research Institute, Albany College of Pharmacy and Health Sciences, Rensselaer, NY 12208, USA

## Error in Figure

In the original publication [1], there was a mistake in the representative image for bFGF + Compound **3**. Specifically, in Figure 5A, there were unintentional mistakes in incorporating representative CAM images from other archived files. The correct image for this panel, along with another PBS control representative image from the same TAT file, is included in the corrected figure. The authors apologize for this unintentional mistake. The changes in the representative images in Figure 5A do not affect the conclusions regarding the biological activity of the compounds studied, as shown in Figure 5B. The images shown are representative single randomly selected images for illustration of the general anti-angiogenesis efficacy of the Tetrac Triazole-PEG conjugated products, and are not for quantitative purposes (5A). The authors state that the scientific conclusions are unaffected. This correction was approved by the Academic Editor. The original publication has also been updated.

## Corrected Figure 5:


